# Global mortality of children after perioperative cardiac arrest: A systematic review, meta-analysis, and meta-regression

**DOI:** 10.1016/j.amsu.2022.103285

**Published:** 2022-02-03

**Authors:** Semagn Mekonnen Abate, Solomon Nega, Bivash Basu, Kidanemariam Tamrat

**Affiliations:** a Department of Anesthesiology, College of Health Sciences and Medicine, Dilla University, Ethiopia; b Department of Internal Medicine, College of Health Sciences and Medicine, Dilla University, Ethiopia; c Department of Anesthesiology, College of Health Sciences and Medicine, Hawassa University, Ethiopia

**Keywords:** Cardiac arrest, Mortality, Incidence, Anesthesia-related

## Abstract

**Background:**

The body of evidence showed that perioperative cardiac arrest and mortality trends varied globally over time particularly in low and middle-income nations. However, the survival of children after cardiac arrest and its independent predictors are still uncertain and a topic of debate. This study was designed to investigate the mortality of children after a perioperative cardiac arrest based on a systematic review of published peer-reviewed literature.

**Methods:**

A comprehensive search was conducted in PubMed/Medline; Science direct, CINHAL, and LILACS from December 2000 to August 2021. All observational studies reporting the rate of perioperative CA among children were included. The data were extracted with two independent authors in a customized format. The methodological quality of the included studies was evaluated using the Newcastle-Ottawa appraisal tool.

**Results:**

A total of 397 articles were identified from different databases. Thirty-eight studies with 3.35 million participants were included. The meta-analysis revealed that the global incidence of perioperative cardiac arrest was 2.54(95% CI: 2.23 to 2.84) per 1000 anesthetics. The global incidence of perioperative mortality was 41.18 (95% CI: 35.68 to 46.68) per 1000 anesthetics.

**Conclusion:**

The incidence of anesthesia-related pediatric cardiac arrest and mortality is persistently high in the last twenty years in low and middle-income countries. This probes an investment in continuous medical education of the perioperative staff and adhering with the international standard operating protocols for common procedures and critical situations.

**Registration:**

This systematic review and meta-analysis is registered in the research registry (UIN: researchregistry6932).

## Highlights

• The incidence of anesthesia-related cardiac arrest and mortality is persistently high in low and middle-income countries.
• The review also showed that anesthesia-related cardiac arrest was very high among younger children with congenital heart disease.
• The overall perioperative cardiac arrest among children has decreased in the last 20 years in high-income countries.
• The Meta-analysis strongly recommends continuous medical education of the perioperative staff, and adherence to the international standard operating protocols.

## 1 Introduction

It is estimated that more than 321.5 million operations were performed worldwide in 2015 [1]. Perioperative care has a tremendous role in treating a broad spectrum of diseases in the alleviation of human suffering at all ages ranges from a neonate for a congenital anomaly to nonagenarians for cataract surgery [1]. However, the quality and safety of perioperative care remain poor particularly in low and middle-income countries where anesthesia-related adverse events were persistent over the years [[2], [3], [4], [5], [6], [7], [8]].

Body of evidence showed that perioperative mortality and anesthesia-related cardiac arrest decreased over time, from 650 per million before the 1970s, to 323 per million in the 1970s–80s, and 143 per million 1990s–2000s in developed countries whereas perioperative mortality and anesthesia-related cardiac arrest remain high among low-income countries [9,10].

Modern anesthesia in otherwise healthy children above one year of age in developed countries has become very safe due to recent advances in pharmacology, intensive education, and training as well as centralization of care while anesthesia in these children in low-income countries is associated with a high risk of mortality due to lack of basic resources and adequate training of health care providers [2,[11], [12], [13], [14], [15], [16]].

There is a regional variation on the rate and types of perioperative anesthetic adverse events in pediatrics. However, plenty of literature reported perioperative pediatric critical incidents which include but are not limited to airway obstruction, respiratory insufficiency, cardiovascular instability, pulmonary edema, hypothermia, hypoxia and desaturation, and delayed awakening which may cause perioperative cardiac arrest and death [2,[17], [18], [19], [2], [20], [21], [22]].

Global anesthesia-related cardiac arrest (CA) and mortality were very low adverse events of perioperative care despite its worst perioperative care and patient outcomes [2]. But the global anesthesia-related cardiac arrest rate may be used as a guide for the quality indicator to improve patient safety in the perioperative period [2,23].

Studies showed that the rate of perioperative cardiac arrest is very high which varied from 2.9 to 4234 cardiac arrests per 10,000 anesthetics and from which 2–60% of cardiac arrests were related to anesthetic management [3,8,12,14,19,22,[24], [25], [26], [27]].

A study in Brazil by Braz et al. showed that the incidence of cardiac arrest was 22.9 per 10000 Anesthesia and 4.58 per 10000 anesthetics were directly related to anesthesia. Meanwhile, general anesthesia, age less than one year, ASA status, emergency surgery, child's clinical condition were the independent predictors of the perioperative cardiac arrest [13].

Another study in the USA by Christensen et al. revealed that 531 children sustained cardiac arrest during over one million anesthetics, and from which 94 of them died. It is also reported that cardiovascular, respiratory, and airway obstructions were the main causes of cardiac arrest respectively [12].

A study from Germany by Hohn et al. reported that there was 25 pediatric cardiac arrest during 36, 243 anesthetics, and twelve of the perioperative cardiac arrests were attributable to anesthesia [25]. Another study from Pakistan by Ahmed et al. among 20216 pediatrics cases reported ten cardiac arrests and four of them were attributable to anesthesia which was related to medication, airway management, and fluid management [28].

The study from China by Gong et al. revealed that there were 104 cardiac arrests and 34 death during 152, 513 anesthetics. Anesthesia was attributable to seven of cardiac arrest which was related to arrhythmia, hypotension, hemorrhage, and high spinal block [29].

The anesthesia database in the USA found out that there were 160 cardiac arrest cases during 217,365 anesthetics. Fourteen of one hundred sixty cardiac arrests were primarily attributable to anesthesia where airway obstruction is accountable for 29% of the cases 70% mortality [30].

Body evidence showed that there is a huge regional disparity in the incidence of mortality and the determinants of perioperative cardiac arrest [5,6,8,10,13,[17], [18], [19],^22^,^24^,^27^,[30], [31], [32], [33], [34], [35], [36], [37], [38], [39]]. Besides, the majority of studies were conducted in developed nations, and data from low and middle-income countries are very scarce.

To the best of our knowledge, there is no meta-analysis conducted so far to address the global survival of children after perioperative cardiac arrest with its prognostic factors such as income level of the nation, the age category of the children, the urgency of surgery, and the presence of comorbidities. Therefore, this Meta-analysis with meta-regression aimed to provide the global pooled incidences of cardiac arrest, mortality, and its independent determinants among children.

## 2 Methods

### 2.1 Protocol and registration

The systematic review and meta-analysis were conducted based on the Preferred Reporting Items for Systematic and meta-analysis (PRISMA) protocols [40], and the Meta-analysis Of Observational Studies in Epidemiology (MOOSE) checklist [41]. This systematic review and meta-analysis is registered in the research registry (UIN: researchregistry6932).

### 2.2 Eligibility criteria

All studies reporting the incidence of perioperative cardiac arrest and mortality among pediatrics receiving any form of anesthetics were included while studies that didn't report the perioperative cardiac arrest and mortality among pediatrics, articles that didn't report full information for data extraction, articles with different outcomes of interest, and Systemic review study design were excluded. The methodological quality of included studies was evaluated with ten points Newcastle-Ottawa appraisal tool as mentioned in the methodological quality assessment section and studies with a methodological score of less than fifty percent were also excluded. The primary outcomes of interest were the incidence of perioperative cardiac arrest and anesthesia-related mortality among pediatrics. The prevalence of risk factors and incidence of overall perioperative mortality were secondary outcomes.

### 2.3 Search strategy

**Fig. 1:**
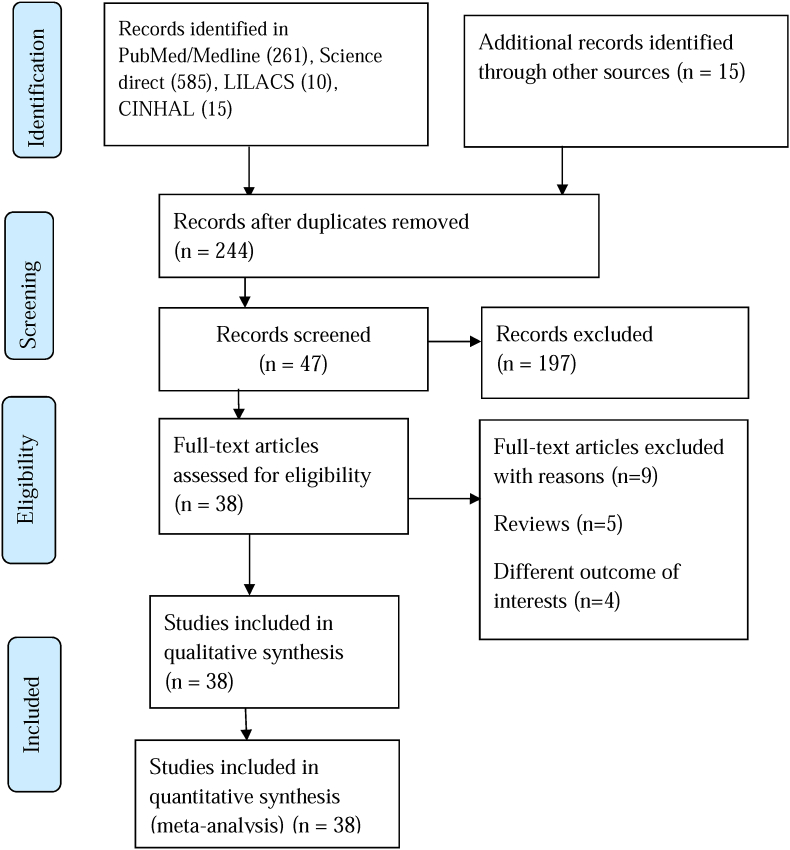
Prisma flow chart.

The search strategy was conducted to explore all available published and unpublished studies reporting perioperative cardiac arrest and mortality among pediatrics from May 2000 to May 2021 without language restrictions. A comprehensive search was employed in this review in different databases. An initial search on PubMed/Medline, Science Direct, CINHAL, and Cochrane Library was carried out followed by an analysis of the text words contained in Title/Abstract and indexed terms. A second search was undertaken by combining free text words and indexed terms with Boolean operators. The third search was conducted with the reference lists of all identified reports and articles for additional studies. Finally, an additional and grey literature search was conducted on Google scholars, and the final search strategy was updated on august 22, 2021. The databases were searched with the following search terms using Pico's (population, interest, context, and design) strategy by combining with AND, OR Boolean operators as pediatrics OR pediatrics OR children OR Child OR neonate OR infant AND perioperative OR in-hospital OR postoperative OR intraoperative OR anesthesia-related AND cardiac arrest OR cardiopulmonary arrest AND mortality OR death OR outcomes AND OR adverse events AND incidence. The final search results were shown with the Prisma flow diagram (Fig. 1).

### 2.4 Data extraction

The data from each study were extracted by SA and BM independently with a customized format excel sheet. The disagreements between the two independent authors were resolved by the other authors. The extracted data included: Author names, country, date of publication, sample size, the incidence of perioperative cardiac arrest, the incidence of anesthesia-related cardiac arrest, mortality, causes of cardiac arrest, and mean age. Finally, the data were then imported for analysis in STATA 16.

### 2.5 Risk of bias assessment

Articles identified for retrieval were assessed by two independent Authors for methodological quality before inclusion in the review using Newcastle-Ottawa appraisal Scale(NOS) (Supplemental Table 1). The disagreements between the Authors appraising the articles were resolved through discussion. Articles with average scores greater than fifty percent were included for data extraction. The methodological quality of this systematic review and meta-analysis was evaluated by a critical appraisal tool for systematic reviews that include randomized or non-randomized studies of healthcare interventions (AMSTAR 2) [42].

### 2.6 Data analysis

Data analysis was carried out in R statistical software version 4.0.2 and STATA 16. The pooled incidence of perioperative cardiac arrest and mortality among pediatrics was determined with a random effect model with restricted maximum likely hood (REML) as there was substantial heterogeneity. The Heterogeneity among the included studies was checked with forest plot, χ2 test, I^2^ test, and the p-values. Substantial heterogeneity among the included studies was investigated with subgroup analysis for categorical moderators (country, surgery, and age group) and meta-regression for continuous covariates (year of publication, mean age, causes of cardiac arrest, and sample size) for outcomes extracted from more than ten studies. Publication bias was checked with a funnel plot and the objective diagnostic test was conducted with Egger's correlation, Begg's regression tests.

## 3 Results

### 3.1 Selection of studies

A total of 2876 articles were identified from different databases with an initial search. Thirty-eight articles were selected for evaluation after the successive screening. Thirty-eight articles with 3.35 million participants were included in the systematic review and Meta-Analysis while nine studies were excluded with reasons (Fig. 1).

### 3.2 Characteristics of included studies

Thirty-eight studies conducted on the incidence of pediatric perioperative and anesthesia-related cardiac arrests with 3.35 million participants were included (Table 1). Nine studies were excluded with reasons. The methodological quality of included studies was moderate to high quality as depicted with the Newcastle-Ottawa Scale Appraisal tool for observational studies (Supplemental Table 1). The majority of included studies didn't report the mean or median age of the study participants but the majority of studies were conducted among children younger than eighteen years in twenty-four studies while seven, two, three, eleven and one studies included children whose age were younger than seventeen, fifteen, sixteen, eleven and one year respectively. Ten of the included studies were conducted in the USA. Sixteen studies were conducted in Germany, Brazil, Pakistan, Nigeria, Thailand, Kenya, South Africa, and Korea two studies in each country while ten studies were conducted in Australia, France, Ethiopia, Iran, Canada, United Kingdom, Ghana, India, Finland, and China. Two prospective multi-country studies were conducted in Europe.

**Table 1:** Epidemiology of included studies.

Author	Year	Country/continent	Cardiac arrest(n)	Sample (N)	Age	quality	Incidence(95%CI) per 1000 anesthetics
Adekola et al. [43]	2015	Nigeria	60	987	<18	10	60.79(45.88, 75.70)
Ahmadi et al. [28]	2012	Iran	59	529	<17	6	111.53(84.71, 138.36)
Ahmed et al. [44]	2008	Pakistan	10	20216	<18	7	0.49(0.19, 0.80)
Ahmed et al. [28]	2009	Pakistan	42	140384	<18	8	0.30(0.12, 0.39)
Bhananker et al. [45]	2007	USA	397	397	<18	10	2.22(1.38, 3.06)
Bharti et al. [46]	2009	India	27	12158	<17	6	–
BRAZ et al. [47]	2006	Brazil	35	15253	<17	8	2.29(1.54, 3.05)
Choi et al. [48]	2014	Korea	30	457529	<18	6	0.07(0.04, 0.09)
Christensen et al. [12]	2018	USA	531	1006686	<17	9	0.53(0.48, 0.57)
Dagan et al. [49]	2018	Australia	211	3781	<18	10	55.81(48.49, 63.12)
Disma et al. [50]	2021	Europe	8	5609	<1	10	1.43(0.44, 2.41)
Ellis et al. [30]	2014	USA	160	217365	<18	6	0.74(0.62, 0.85)
Flick et al. [30]	2007	USA	27	92881	<17	10	0.29(0.18,0.40)
Gerrit et al. [51]	2020	German	18	22650	<18	10	0.79(0.43, 1.16)
Gong et al. [29]	2018	China	104	125513	<18	8	0.83(0.67, 0.99)
Gonzalez et al. [24]	2014	Brazil	22	10649	<18	10	2.07(1.20,2.93)
Habre et al. [52]	2017	Europe	9	30874	<18	10	0.29(0.10, 0.48)
Hohn et al. [25]	2019	German	29	36243	<17	10	0.80(0.51,1.09)
Islam et al. [53]	2021	Kenya	2	83	<18	5	24.10(-8.89, 57.09)
Lee et al. [6]	2016	Korea	42	49373	<18	8	0.85(0.59,1.11)
Lync et al. [54]	2011	Canada	4	129	<17	6	31.01(1.10, 60.92)
Menga et al. [55]	2015	USA	11	2524	<18	8	4.36(1.79, 6.93)
Meyer et al. [56]	2017	South Africa	47	8325	<18	8	5.65(4.04, 7.26)
Morray et al. [33]	2000	USA	289	289	<18	6	0.33(0.10, 0.56)
Murat et al. [19]	2004	France	8	24165	<18	8	–
Newland et al. [57]	2002	USA	144	72959	<18	10	1.97(1.65, 2.30)
Newton et al.[58]	2020	Kenya	185	6005	<18	10	1.97(1.65, 2.30)
Nutchanart et al. [59]	2009	Thailand	150	25098	<15	8	5.98(5.02, 6.93
Peiffer et al. [60]	2018	Ghana	8	468	<18	10	5.98(0.74, 11.04)
Ramamoorthy et al. [61]	2010	USA	372	372	<18	8	–
Siriphuwanun et al. [62]	2014	Thailand	721	44339	<18	10	16.26(15.08(17.44)
Skellett et al. [63]	2020	UK	1580	110705	<15	10	14.27(13.57, 14.97)
Sprung et al. [27]	2003	USA	223	518294	<18	8	0.43(0.37, 0.49)
Suominen et al. [64]	2001	Finland	82	1115	<16	6	73.54(58.22, 88.86)
Talabi et al. [65]	2018	Nigeria	64	4040	<15	8	15.84(11.99, 19.69)
Tarekegn et al. [66]	2021	Ethiopia	5	849	<11	10	5.89(0.74, 11.04)
Torborg et al. [67]	2018	South Africa	12	2024	<16	10	5.93(2.58, 9.27)
Zgleszewski et al. [22]	2016	USA	142	276209	<18	10	0.51(0.43, 0.60)

All of the included studies reported incidence of perioperative cardiac arrest while only thirty-four of them reported perioperative mortality. Twenty-one of the included studies reported perioperative cardiac arrest which was primarily attributable to anesthesia while anesthesia-related mortality was reported in fifteen studies.

The majority of included studies reported the perioperative causes of cardiac arrest including but not limited to hypotension, hemorrhage, airway obstruction, hypoxia, pulmonary edema, arrhythmias, sepsis, medication, equipment failure, children's critical condition, ASA status, emergency surgery, hypothermia, and professional expertise in pediatric anesthesia management. The majority of the included studies were conducted based on data extracted from national and/or hospital perioperative cardiac arrest registries while the rest were prospective observational studies.

## 4 Meta-analysis

### 4.1 Perioperative cardiac arrest and mortality

**Fig. 2:**
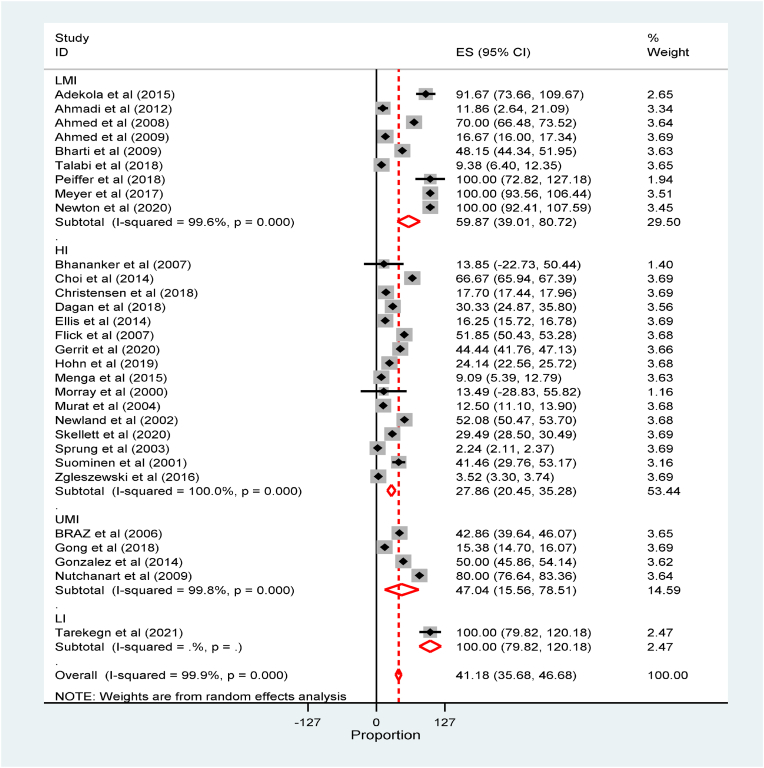
Forest plot for the global incidence of perioperative Mortality among children per 1000 anesthetics: The midpoint of each line illustrates the rate; the horizontal line indicates the confidence interval, and the diamond shows the pooled incidence of mortality.

The meta-analysis revealed that the global incidence of perioperative cardiac arrest was 2.54(95% CI: 2.23 to 2.84, 34 studies, 3.35 million participants) per 1000 anesthetics (Supplemental Fig. 1). The incidence of perioperative cardiac arrest was the highest among low and middle-income countries (Supplemental Fig. 2). The global incidence of perioperative mortality was 41.18(95% CI: 35.68 to 46.68, studies, 3.2million participants) per 1000 anesthetics (Fig. 2).

**Fig. 3:**
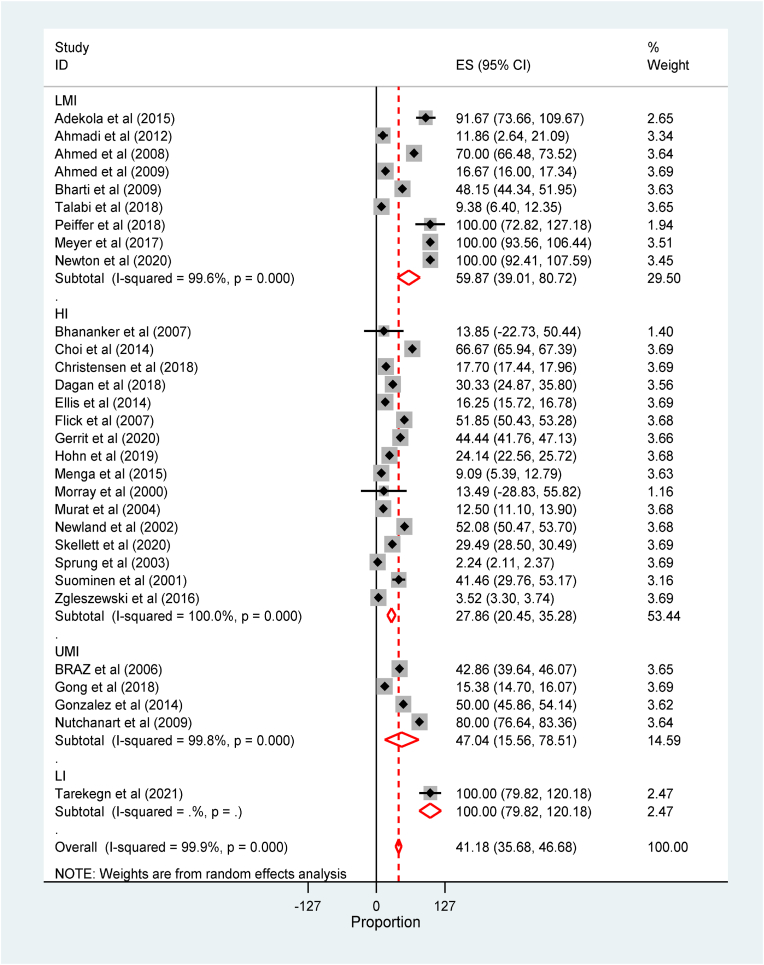
Forest plot for subgroup analysis of the global incidence of perioperative Mortality among children by countries economic level per 1000 anesthetics. The midpoint of each line illustrates the rate; the horizontal line indicates the confidence interval, and the diamond shows the pooled incidence of mortality.

Subgroup analysis was conducted among the income level of countries using the recent world bank classification of countries by economic level [68]. The subgroup analysis by countries economic level showed that the global incidence of perioperative mortality among children was the highest among low-income countries as compared to high and upper-middle-income countries 27.86(95% CI:20.45 to 35.28) and 47.04(95% CI: 15.56 to 78.51) respectively per 1000 anesthetics (Fig. 3) while the perioperative incidence of mortality among children by age category revealed that mortality was the highest in children younger than one year and 1–5 years(Supplemental Fig. 3).

### 4.2 Anesthesia-related cardiac arrest

**Fig. 4:**
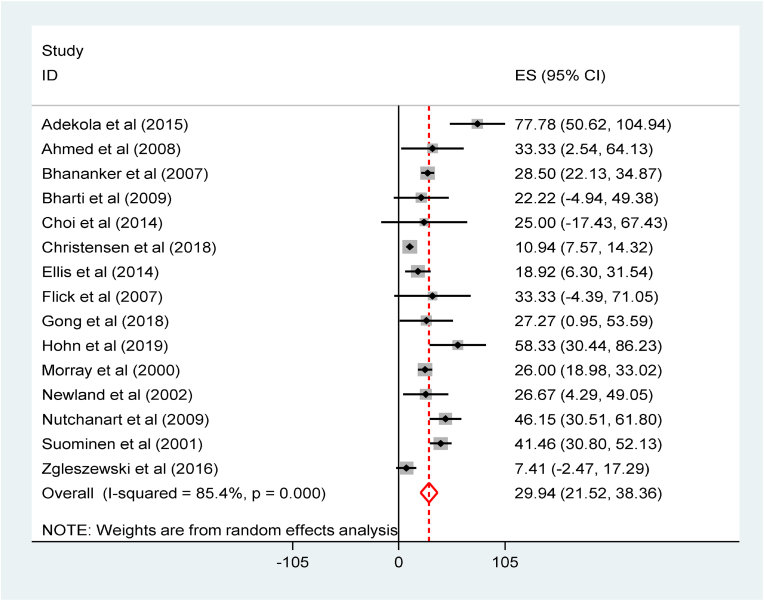
Forest plot for the incidence of anesthesia-related cardiac arrest among children per 1000 anesthetics: The midpoint of each line illustrates the rate; the horizontal line indicates the confidence interval, and the diamond shows the pooled incidence of anesthesia related-cardiac arrest.

The incidence of anesthesia-related cardiac arrest was reported in twenty-one studies which were estimated from patients having perioperative anesthesia-related cardiac arrest among children receiving anesthetics. The meta-analysis showed that the incidence of anesthesia-related cardiac arrest was 27.68(95% CI: 17.92 to 37.44, 21 studies, 30.6 million participants) (Fig. 4). The subgroup analysis of anesthesia-related cardiac arrest by age category revealed that the incidence of anesthesia-related cardiac arrest was higher among children younger than one year 4.23(95% CI: 2.49 to 5.95) and 1–5 years 3.78(95% CI: 0.05 to 7.51) category as compared to 5–10 years 1.05(95% CI: 0.15 to 1.97), and >10 years old 1.04(95% CI: 0.20 to 1.88) per 1000 anesthetics respectively (Supplemental Fig. 4).

### 4.3 Anesthesia-related mortality

**Fig. 5:**
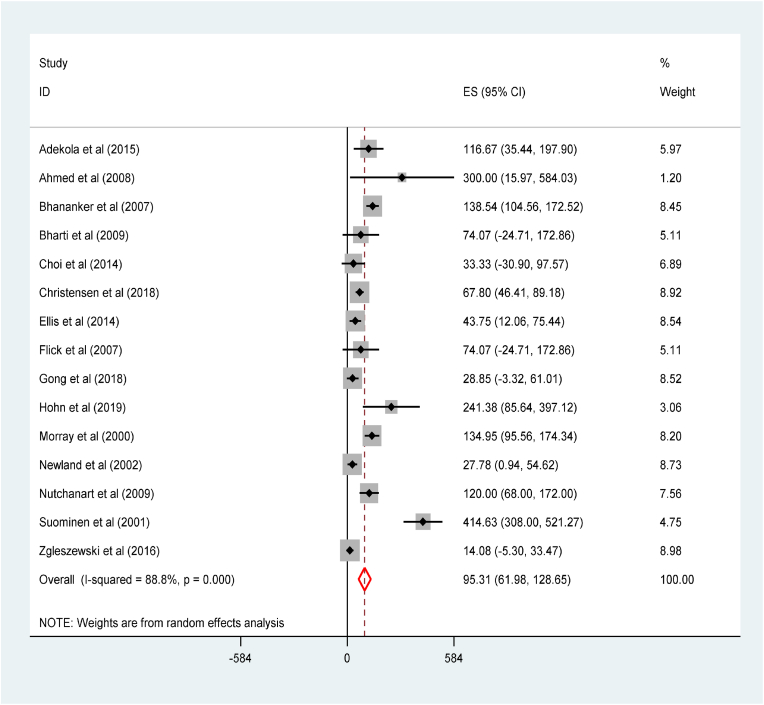
Forest plot for the incidence of anesthesia-related mortality among children per 1000 anesthetics: The midpoint of each line illustrates the rate; the horizontal line indicates the confidence interval, and the diamond shows the pooled incidence of mortality.

The incidence of anesthesia-related mortality among children was 95.31(95% CI: 61.98 to 128.65) per 1000 anesthetics (Fig. 5). The subgroup analysis of the incidence of anesthesia-related mortality among children by their age categories revealed that the lowest was in children older than ten years 0.09(95% CI: 0.02 to 0.16) while the highest was in children younger than one-year-old 1.01(95% CI:0.33 to 1.69) (Supplemental Fig. 5). Besides, the anesthesia-related mortality was the highest among low middle-income countries 131.55(95% CI: 101.83 to 161.26) as compared to high-income countries 94.70(95% CI: 54.69 to 134.71) (Supplemental Fig. 6). However, no anesthesia-related mortality was reported among low-income countries.

### 4.4 Meta-regression

The meta-analysis showed that there is substantial heterogeneity between the included studies as depicted with I-squared statistics and its corresponding p-value. Therefore, subgroup analysis was done for anesthesia-related cardiac arrest and mortality with categorical moderators including income level of the countries, age of categories of children as younger than one-year-old, 1–5 years, 5–10 years, and >10 years old.

We also conducted a weighted meta-regression to investigate the sources of heterogeneity between the included studies with continuous covariates sample size, and year of publication. Besides, the meta-regression is intended to explore the correlation and change in the time trends of perioperative cardiac arrest, overall mortality, anesthesia-related cardiac arrest, and anesthesia-related mortality.

**Fig. 6:**
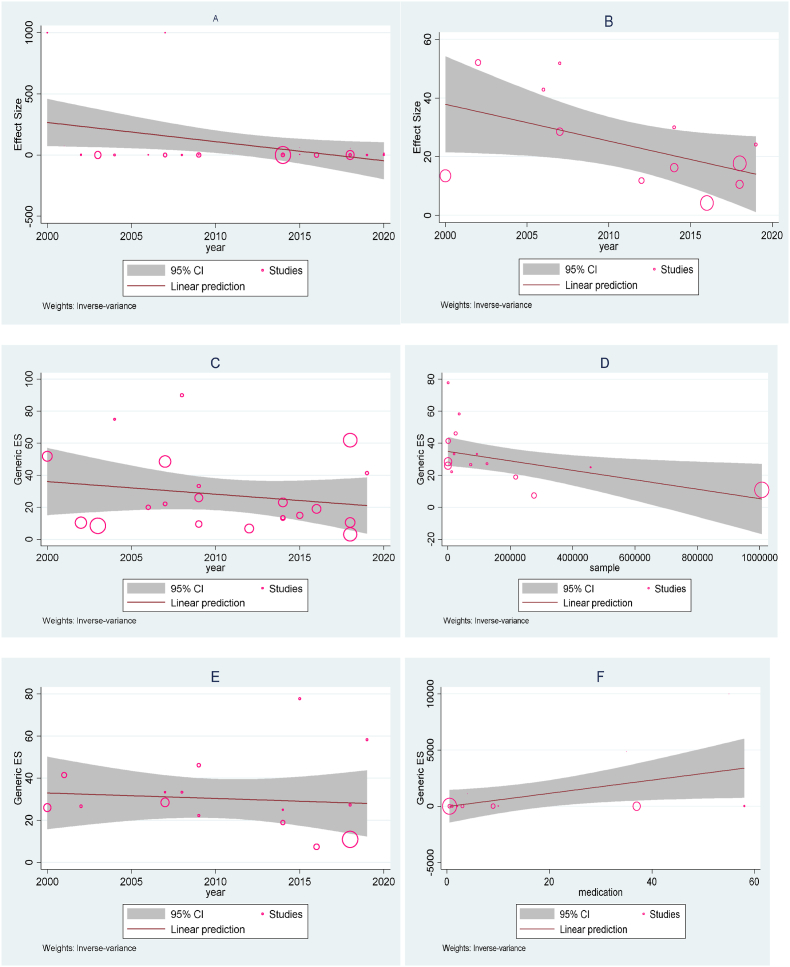
Bubble plot for meta-regression analysis: A: perioperative cardiac arrest; B: perioperative mortality; C: Anesthesia-related cardiac arrest; D: Anesthesia-related mortality; E: Anesthesia-related mortality and F: cardiac arrest with medication error.

The meta-regression showed that pediatrics perioperative cardiac arrest had strong correlation with year of publication where CA decrease from 2000 to 2020 (slope = −15.64(95% CI:-30.27 to −1.003, p = 0.036 Fig. 6A). The overall perioperative mortality also decrease from 2000 to 2020(slope = −1.25 95% CI:-2.53 to 0.02, p = 0.05, Fig. 6B).

The weighted meta-analysis didn't show significant difference on perioperative anesthesia-related cardiac arrest and mortality among pediatric population receiving anesthetics (slope = −0.79, 95% CI:-2.52 to 0.94, p = 0.37, Fig. 6C and slope = - 0.26, 95% CI: 1.96 to 1.16, p = 0.72, Fig. 6E) respectively. However, there was a strong relation between perioperative anesthesia related mortality and sample size (slope = 58.95, 95% CI: 2.52 to 115.37, p = 0.041, Fig. 6F).

We planned to conduct a meta-regression with a mean age of participants but the number of studies reporting the median or mean age of the participants was less than ten where the meta-regression assumption will not be satisfied and that is why we didn't run meta-regression.

The meta-regression was also conducted to investigate the correlation between sample size contribution of individual studies on perioperative cardiac arrest, perioperative mortality, and anesthesia-related mortality. However, none of them showed a significant differences (slope = −0.00003, 95% CI: 0.00006 to 0.00007, p = 0.125), (slope = −0.00004, 95% CI: 0.00009 to 0.00001, p = 0.11), and (slope = −0.79, 95% CI: 2.52 to 0.94, p = 0.37) respectively.

### 4.5 Determinants of perioperative CA

**Fig. 7:**
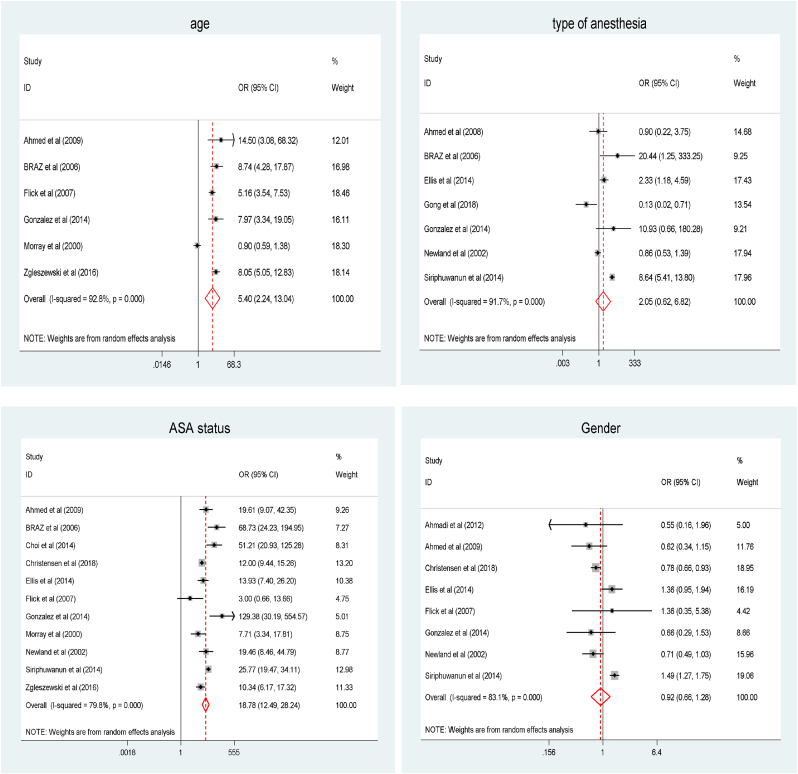
Forest plot for factor analysis for acute myocardial injury among patients with COVID-19: The midpoint of each line illustrates the prevalence; the horizontal line indicates the confidence interval, and the diamond shows the pooled odds ratio.

We performed factor analysis to investigate the determinants of perioperative CA among pediatrics. The pediatrics who had ASA status >3 were almost 19 times more likely to experience perioperative CA relative to those with ASA status <3, OR = 18.78(95% CI: 12.48 to 28.24). In addition, pediatrics who were younger than 1 year old were 5 times more likely to develop perioperative CA, OR = 5.40(95% CI: 2.24 to13.04) (Fig. 7).

### 4.6 Publication bias

**Fig. 8:**
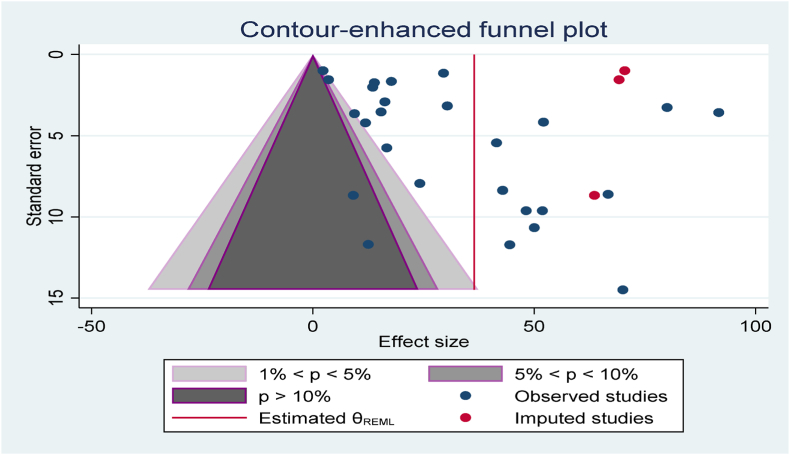
Contour-enhanced funnel plot and trim fill to assess publication bias. The vertical line indicates the effect size whereas the diagonal line indicates the precision of individual studies with a 95% confidence interval.

The publication bias was investigated with funnel plot asymmetry and egger's regression, Begg's rank correlation test, and trim fill method. Neither the egger's regression nor Begg's rank correlation test showed significant difference (p = 0.056 and p = 0.128 respectively. However, the trim fill showed that three studies were missed (Fig. 8).

## 5 Discussion

To the best of our knowledge, this is the first systematic review, a meta-analysis with meta-regression on perioperative anesthesia-related cardiac arrest and mortality in a global context among children receiving anesthetics. The current study was intended to investigate the overall perioperative cardiac arrest and mortality, anesthesia-related cardiac arrest and mortality, and its determinants among children receiving anesthetics.

This systematic review revealed that the incidence of perioperative cardiac arrest was 2.54 per 1000 anesthetics which is lower than the reports of included studies and a meta-analysis of perioperative mortality conducted in brazil [2,3,8,12,15,19,22,24,25,[28], [29], [30],[43], [44], [45], [46], [47], [48], [49],^54^,^55^,^57^,^59^,^62^,^63^,^65^,^67^]. This discrepancy might be explained by the inclusion of many studies with a large sample from different countries globally.

The incidence of perioperative mortality was the highest in low-income, lower-middle-income and upper middle-income countries as compared to high income-countries which is consistent with included studies and a meta-analysis by Braz et al. in Brazil [2,4,16,24,31,[69], [70], [71]]. The high incidence of perioperative cardiac arrest and mortality in low and middle-income countries might be because of a lack of standard perioperative monitors, emergency medications, equipment, adequate preoperative evaluation and preparation, adequate training of healthcare workers [16,22,39,[69], [70], [71]].

This study revealed that perioperative cardiac arrest and mortality was higher among children younger than one-year-old which is consistent with other studies conducted around the globe where mortality was very high among younger children, and children with congenital heart disease, ASA status 3 and above, and emergency surgeries [27,35,36,61,64,71,72].

The meta-regression by the year of publication showed that both perioperative cardiac arrest and mortality decreased over time which is consistent with a meta-analysis study of perioperative cardiac arrest in Brazil [2].

The meta-analysis showed that anesthesia-related cardiac arrest and mortality were very high and consistent with the included studies. However, anesthesia-related cardiac arrest and mortality didn't change over time as depicted with meta-regression by year of publication, unlike perioperative cardiac arrest and mortality which declines over time. This finding is in line with a meta-analysis and meta-regression by Koga et al. among low and high-income countries among all surgical patients [69].

We conducted a factor analysis to identify the independent predictors of perioperative cardiac arrest. The meta-analysis showed that children who are younger than 1 year old, ASA physical status >3, and general anesthesia were the independent predictors of perioperative cardiac arrest which is comparable to a meta-analysis by Braz et al. among trauma patients [73].

Children with the ASA physical status >3 was nearly 19 times more likely to develop perioperative cardiac arrest as compared to ASA physical status <3 which may be related to a lack of expertise in pediatrics anesthesia, and resources, particularly in developing nations. The age of pediatrics has also increased the odds of perioperative cardiac arrest by five times compared to older children. However, the gender of the patient didn't show any significant difference in the incidence of perioperative cardiac arrest and mortality.

### 5.1 Quality of evidence

The methodological quality of included studies was moderate to high quality as illustrated with the Newcastle-Ottawa scale appraisal tool for meta-analysis of observational studies. However, substantial heterogeneity associated with dissimilarities of included studies in the age group, and sample size could affect the overall quality of evidence.

### 5.2 Implication for practice

Body of evidence revealed that perioperative cardiac arrest and mortality decline over time. But, anesthesia-related cardiac arrest and mortality are still high and consistent over time, particularly in low and middle-income countries. Therefore, a mitigating strategy is required by different stakeholders to prevent and manage anesthesia-related cardiac arrest and mortality.

### 5.3 The implication for further research

The meta-analysis revealed that the rate of anesthesia-related cardiac arrest and mortality is very high. However, the included studies were too heterogeneous and retrospective studies were also associated with selection and reporting biases. Therefore, further prospective observational and randomized controlled trials are required to provide a firm conclusion.

### 5.4 Limitation of the study

The meta-analysis included studies with moderate to high methodological quality and large sample size. However, the majority of included studies didn't report data on anesthesia-related mortality, determinants, and mean age to investigate the independent predictors in which case it would be difficult to provide conclusive evidence.

## 6 Conclusion

This meta-analysis showed that the incidence of anesthesia-related cardiac arrest and mortality among children was very high among low and middle-income countries which are persistent in the last two decades. Besides, children whose age was younger than one year, ASA status >3 congenital heart disease, and emergency surgery were independent predictors of perioperative cardiac arrest and mortality. This dictates a mitigating strategy by stakeholders to prevent and manage children from perioperative cardiac arrest and mortality.

## Ethics approval and consent to participate

Not applicable.

## Consent for publication

Not applicable.

## Availability of data and materials

Data and material can be available where appropriate.

## Provenance and peer review

Not commissioned, externally peer-reviewed.

No funding was obtained from any organization.

## Authors' contributions

SA and SN conceived the idea design of the project. SA, BB, SN, and KT were involved in searching strategy, data extraction, quality assessment, analysis, and manuscript preparation. All authors read and approved the manuscript.

## Declaration of competing interest

The authors declare that there are no competing interests.
