# Supplementary material for: Global mortality of children after perioperative cardiac arrest: A systematic review, meta-analysis, and meta-regression
Source: Ann Med Surg (Lond). 2022 Feb 3;74:103285. doi: 10.1016/j.amsu.2022.103285 (PMC8858756; doi:10.1016/j.amsu.2022.103285)
Supplement: Multimedia component 1 [file mmc1.docx]

List of supplementary figures

**Supplemental Fig 1:** Forest plot for the global incidence of perioperative cardiac arrest among children in 1000 anesthetics: The midpoint of each line illustrates the rate; the horizontal line indicates the confidence interval, and the diamond shows the pooled incidence of perioperative cardiac arrest.

**Supplemental Fig 2**: Forest plot for subgroup analysis of the global incidence of perioperative cardiac arrest by income level of countries. The midpoint of each line illustrates the rate; the horizontal line indicates the confidence interval, and the diamond shows the pooled incidence of perioperative cardiac arrest

**Supplemental Fig 3:** Forest plot for the subgroup analysis of incidence of perioperative mortality by age category among children: The midpoint of each line illustrates the rate; the horizontal line indicates the confidence interval, and the diamond shows the pooled incidence of perioperative cardiac arrest

**Supplemental fig 4:** Forest plot for the subgroup analysis of incidence of anesthesia related cardiac arrest by age category among children: The midpoint of each line illustrates the rate; the horizontal line indicates the confidence interval, and the diamond shows the pooled incidence of anesthesia-related cardiac arrest

**Supplemental fig 5:** Forest plot for subgroup analysis of incidence of anesthesia related mortality among children per 1000 anesthetics by age among children: The midpoint of each line illustrates the rate; the horizontal line indicates the confidence interval, and the diamond shows the pooled incidence of anesthesia-related mortality.

 **Supplemental fig 6:** Forest plot for subgroup analysis of incidence of anesthesia related mortality among children per 1000 anesthetics by income level of countries among children: The midpoint of each line illustrates the rate; the horizontal line indicates the confidence interval, and the diamond shows the pooled incidence of anesthesia-related mortality.
