# Supplementary material for: Global mortality of children after perioperative cardiac arrest: A systematic review, meta-analysis, and meta-regression
Source: Ann Med Surg (Lond). 2022 Feb 3;74:103285. doi: 10.1016/j.amsu.2022.103285 (PMC8858756; doi:10.1016/j.amsu.2022.103285)
Supplement: Multimedia component 2 [file mmc2.docx]

**Supplemental Table 1:** Methodological quality of included studies as per Newcastle-Ottawa scale

| Study | Selection | | | | Comparability | Outcome | | Quality Score |
| --- | --- | --- | --- | --- | --- | --- | --- | --- |
|  | Representativeness of the sample | Sample size calculation | Non-respondents | Ascertainment of confounders | Confounding factor controlled | Assessment of outcomes | Statistical tests |  |
| Adekola et al | 1 | 1 | 1 | 2 | 2 | 2 | 1 | 10 |
| Ahmadi et al | 1 | 1 | 1 | 0 | 0 | 1 | 1 | 6 |
| Ahmed et al | 1 | 1 | 1 | 0 | 2 | 1 | 1 | 7 |
| Ahmed et al | 1 | 1 | 1 | 2 | 0 | 2 | 1 | 8 |
| Bhananker et al | 1 | 1 | 1 | 2 | 2 | 2 | 1 | 10 |
| Bharti et al | 1 | 1 | 1 | 0 | 0 | 1 | 1 | 6 |
| BRAZ et al | 1 | 1 | 1 | 2 | 0 | 2 | 1 | 8 |
| Choi et al | 1 | 1 | 1 | 0 | 0 | 2 | 1 | 6 |
| Christensen et al | 1 | 1 | 1 | 2 | 2 | 2 | 1 | 9 |
| Dagan et al | 1 | 1 | 1 | 2 | 2 | 2 | 1 | 10 |
| Disma et al | 1 | 1 | 1 | 2 | 2 | 2 | 1 | 10 |
| Ellis et al | 1 | 1 | 1 | 0 | 0 | 2 | 1 | 6 |
| Flick et al | 1 | 1 | 1 | 2 | 2 | 2 | 1 | 10 |
| Gerrit et al | 1 | 1 | 1 | 2 | 2 | 2 | 1 | 10 |
| Gong et al | 1 | 1 | 1 | 2 | 0 | 2 | 1 | 8 |
| Gonzalez et al | 1 | 1 | 1 | 2 | 2 | 2 | 1 | 10 |
| Habre et al | 1 | 1 | 1 | 2 | 2 | 2 | 1 | 10 |
| Hohn et al | 1 | 1 | 1 | 2 | 2 | 2 | 1 | 10 |
| Islam et al | 0 | 1 | 1 | 0 | 0 | 2 | 1 | 5 |
| Lee et al | 1 | 1 | 1 | 1 | 2 | 0 | 1 | 8 |
| Lync et al | 1 | 1 | 1 | 0 | 0 | 2 | 1 | 6 |
| Menga et al | 1 | 1 | 1 | 0 | 0 | 2 | 1 | 8 |
| Meyer et al | 1 | 1 | 1 | 2 | 0 | 2 | 1 | 8 |
| Morray et al | 1 | 1 | 1 | 0 | 0 | 2 | 1 | 6 |
| Murat et al | 1 | 1 | 1 | 2 | 0 | 2 | 1 | 8 |
| Newland et al | 1 | 1 | 1 | 2 | 2 | 2 | 1 | 10 |
| Newton et al | 1 | 1 | 1 | 2 | 2 | 2 | 1 | 10 |
| Nutchanart et al | 1 | 1 | 1 | 2 | 2 | 0 | 1 | 8 |
| Peiffer et al | 1 | 1 | 1 | 2 | 2 | 2 | 1 | 10 |
| Ramamoorthy et al | 1 | 1 | 1 | 2 | 0 | 2 | 1 | 8 |
| Siriphuwanun et al | 1 | 1 | 1 | 2 | 2 | 2 | 1 | 10 |
| Skellett et al | 1 | 1 | 1 | 2 | 2 | 2 | 1 | 10 |
| Sprung et al | 1 | 1 | 1 | 2 | 0 | 2 | 1 | 8 |
| Suominen et al | 1 | 1 | 1 | 2 | 0 | 0 | 1 | 6 |
| Talabi et al | 1 | 1 | 1 | 2 | 0 | 2 | 1 | 8 |
| Tarekegn et al | 1 | 1 | 1 | 2 | 2 | 2 | 1 | 10 |
| Torborg et al | 1 | 1 | 1 | 2 | 2 | 2 | 1 | 10 |
| Zgleszewski et al | 1 | 1 | 1 | 2 | 2 | 2 | 1 | 10 |
